# A Treatise on the Injuries, the Diseases, and the Distortions of the Spine, &c. &c.

**Published:** 1832-07-01

**Authors:** 


### Medico-Chirurgical Review,

### No. XXXIII.

APRIL 1, TO JULY 1, 1832.

### I.

A Treatise on the Injuries, the Diseases, and the Distortions of the Spine; founded on an Essay to which the Jacksonian Prize, for the Year 1826, was adjudged by the Royal College of Surgeons. By *R. A. Stafford*, Surgeon to the St. Mary-le-bone Infirmary, &c. Octavo, pp. 302. London, 1832.

Mr. STAFFORD informs us, that his information on the subjects of which he professes to treat has chiefly been acquired at the bedside, from cadaveric investigations, or from morbid preparations. His arrangement is that proposed by the Jacksonian Committee of the College—first, the Congenital Diseases of the Spine—secondly, the Injuries—thirdly, the Diseases and Distortions of the Vertebræ—and lastly, those of the Medulla and its Membranes. Some of these subjects have been copiously discussed of late years, and little novelty can reasonably be expected from a fresh inquirer. Others, for instance the congenital malformations, are not likely, we suspect, to shine with any new or brilliant lights, whoever he may be that ventures to bring them into public view. Perhaps a surgeon of great experience might communicate facts of which we are ignorant, and from them draw conclusions practically useful, respecting the injuries to which the spinal column is liable; but we doubt whether any young man can possibly do what Mr. Stafford confidently promises—compose a work on the diseases and injuries of the spine, which shall not be, for the most part, a compilation. We do not utter this opinion to derogate in the least from Mr. Stafford's merit, but we make the prediction with too firm an expectation, though not with any wish, of seeing it fulfilled. We shall pass without further preface to the examination of the volume.

For the various malformations of the spine, Mr. Stafford particularly refers to the work of M. Ollivier. The first chapter, then, is on spina bifida, and, as it would be no disgrace to Mr. Stafford to pretend to no extended experience on a malformation comparatively rare, we find the best authorities freely quoted. As we have not space, however, for the opinions of those older writers, to whom diligent students and zealous inquirers can always refer, we shall pick out what new matter chances to present itself, and leave the remainder for the reader of the volume. The following short extracts shew the seat of spina bifida, which is by no means confined to the lumbar region.

"We now come to the seat of Spina Bifida; it has been usually described as a lumbar tumor, but it may occur in any part of the spinal column, from the cervical portion to that of the sacrum, and various authors relate well-authenticated cases where each part, and sometimes the whole of the canal, has been defective and involved in the disease. It is not necessary, however, to cite these cases; we have examples sufficient of a later date, and our museums teem with preparations illustrative of this fact. In some we see the whole spine defective in its spinous processes and rings; in others, two parts of the column are malformed, leaving the intermediate space perfect, and in others again, the cervical, the dorsal, lumbar, and the sacral. The cause of this imperfection of the vertebræ must be no doubt owing to the cessation of ossification, and to the imperfect closure of their rings during utero-gestation, and this is precisely analogous to all those malformations which originate from the want of union of the two halves of the fœtus while in progress of formation; such as a portion of the back part of the occipitis being deficient, cleft palate, hare-lip, and an incomplete junction of the parietes of the abdomen at the linea alba. Spina Bifida is often combined with one or other of these anomalies, and there is a preparation in the College of Surgeons which well illustrates the analogy between them. In the same fœtus, there is spina bifida in the dorsal region, and an incomplete closure of the parietes of the abdomen, and thus the peritoneum and the intestines protrude." 12.

"This fluid is most frequently contained between the arachnoid membrane and pia mater, but occasionally it has been known to exist between the arachnoid membrane and dura mater, and sometimes between both at the same time. Portal, with others, speak of its having been contained in a canal which they all assert exists in the substance of the medulla; but the correctness of this fact is doubted by anatomists. In all those cases which were drawn on purpose to represent the distribution of the nerves, the fluid was contained between the arachnoid membrane and pia mater; but in drawing I. it existed in separate cells or cysts. In this case, when the tumor was punctured, all the fluid was not evacuated at once, but there remained behind small distended bladders of it, unconnected with the common cyst. This was seen more particularly in one on the right side, which being itself about the size of an orange, was obliged to be punctured, to let out its contents. Upon examining the tumor after death, there were found two large cysts, and a number of smaller ones, about the size of a pea, existing in the parietes of the tumor. The large cavity in drawing No. I. was no doubt the common cyst connected with the vertebral canal, with its neck, or the part joining immediately with the spine, filled up by newly-organized lymph, and thus closing up the communication. The smaller one of the two was quite impervious, and its internal surface was smooth; it appeared to be continuous with the external membrane, or what would have been the dura mater of the other cyst; and it is probable that it had been connected originally with it, and afterwards closed up, and then, if this conjecture be right, the fluid must have escaped through the arachnoid membrane and dura mater. The other cells also were quite impermeable, and it would be almost impossible to account for their existence." 18.

In order to appreciate the value of any proposed plan of treatment, as well as to avoid rash measures on his own part, the surgeon should be aware of the condition and disposition of the spinal nerves in this complaint.

"In spina bifida it appears that either part or all the filaments which form the posterior spinal nerves, corresponding to the solution of continuity of the vertebral canal, are distributed upon the internal walls of the tumour, and that they generally terminate there. These arise from the cord in various ways; sometimes only by single filaments, which pass out of the aperture of the canal through the fluid contained in the tumor, unattached to any thing until they arrive at its parietes, where they are dispersed. This is particularly well illustrated by the drawing,* No. V. fig. 1; here we see two of the nerves arising and distributing themselves in this manner. Sometimes, again, the posterior spinal nerves come out of the opening in the canal, and instead of going straight through the fluid, pass round the internal surface of the tumor, and thus form a network of nervous filaments. Burgius mentions a case of this description, where the internal surface of the tumor was so surrounded by nervous columns and fibres, that it resembled the internal structure of the auricles and ventricles of the heart. In other cases there arises a kind of peduncle in the middle of the tumor, of a nervous mass, resembling a mushroom, of which the stalk is formed of filaments of nerves bound together by cellular tissue, and the head by their extremities spread out and ending upon the upper and internal surface of the tumor. Brunner has particularly taken notice of this variety of distribution; and from the drawing,† No. V. fig. 2, one would almost imagine he was alluding to the same case.

The posterior spinal nerves, which are distributed upon the internal walls of the tumor, do not always pass through the aperture connected with the vertebral canal; but sometimes they pierce as it were the tumor, and then distribute themselves on the internal surface. There are two preparations of this description in Mr. Llangstaff's Museum; in the one they pierce the tumor at its back part, and pass straight through the fluid; in the other they also pierce the back part of the tumour, but they immediately curl round its walls.

There appears to be some degree of regularity of the distribution of the nerves when spina bifida occurs at the upper part of the canal; for here the posterior nerves are only involved in the disease, whilst, at the lower portion, the whole cord is continually influenced by it. Thus we see in some cases only a few nervous filaments erring in their course, while in others we find the whole bundle of nerves, which form the cauda equina, going out of the canal and attaching themselves to the top of the tumor, and returning into it again, as may be observed in drawing‡ V. fig. 3.

Sometimes, again, we find that the whole of the medulla is brought out of its course through the aperture of the canal, and that it completely ends in the tumor, leaving the canal below quite hollow. Various authors, among whom are Brunner, Hoin, Apinus, and Sandifort, relate cases of this description; and, no doubt, if the owner of the preparation from which the drawing V. fig. 2, was taken, would permit a farther dissection to be made of it, we should find the canal of the sacrum quite hollow, and that the peduncle in the middle, and the nerves around, was the whole of the cauda equina terminating in the tumor; for it so exactly resembles that case before mentioned, described by Brunner, that one naturally would expect to find the same result.

It does not appear, however, that the medulla, or the lower part of the canal, always deviates from its course, or that the nerves are altered in their distribution; for it may occasionally be seen that no alteration whatever takes place, and that neither itself nor its nerves are changed either in their arrangement or in their structure. This is exemplified by drawing V. fig. 4.§ Here we see the bundle of nerves, forming the cauda equina, following their usual course.

The medulla spinalis also in spina bifida, at the part where the malformation of the canal occurs, becomes changed in its structure in various ways; sometimes it has been found inflamed—sometimes not so large as natural—and sometimes

---

* Vide Drawing in the Library of the College of Surgeons, and Preparation, Museum St. Bartholomew's Hospital.

**of a firmer consistence; sometimes, again, it has been rendered completely soft —and at other times has been found altogether deficient."** 25.

The remedial means proposed or adopted for this formidable complaint have been, ligature—seton—compression—and puncture with compression. The two former were proposed by Mr. B. Bell and Dessault; there are many obvious and serious objections to their employment. The two latter were recommended by Mr. Abernethy, and have frequently been employed. Compression is a palliative measure, and has proved no more. Puncture, with compression, appears to promise rather more, but we must not expect much from any treatment. A short time ago we saw a young girl, apparently, if our memory serves us, some fourteen years of age, who had had spina bifida since infancy. The tumour was in the sacral region, about the size of an orange but flatter, with thin but not inflamed parietes. The girl was in good health, though of strumous appearance, and seemed to suffer little inconvenience from the disease. We believe that compression and puncture had been tried, but latterly all applications were abandoned, and the tumour was not on the increase. We shall now mention some cases related by Mr. Stafford, as they will convey more information on such a point than descriptions.

CASE 1. *Dec.* 10, 1826. Mary Cotterell, aged 11 days, has a large swelling situated over the whole of the posterior surface of the sacrum. It is 14 inches in circumference, tense, elastic, evidently containing fluid. The integuments on the right side are very much inflamed, and at several parts of it, points of ulceration have commenced. The child is healthy and seems to suffer little. The swelling was one-third less at birth, and has been daily increasing.

As the fluid must soon have escaped from the progress of the ulceration, the tumour was punctured with a needle. By the next day a few drops only of fluid had escaped, and a small opening was therefore made with a lancet, and about two ounces of straw-coloured serum drawn off. Sticking-plaster was applied over the opening. 14th. Tumour again punctured, and nearly three ounces of fluid discharged. 15th. Plaster removed, and two ounces allowed to escape—redness diminished, and integuments beginning to contract. 18th. Tumour more distended, and its parietes considerably thickened; two openings made, and a little blood discharged. 20th. Swelling almost as great as at first; small trocar introduced, and 4 ounces of bloody serum drawn off; parietes of tumour found very much thickened; child restless. 22d. Tumour less inflamed and distended; four ounces of thicker fluid, mixed apparently with pus, drawn off by trocar; two or three strips of plaster applied tightly over swelling, with roller round body. Child easier since 20th. 26th. Improved; tumour evidently decreased, and parietes thickening; almost all the fluid evacuated to day; it is fetid, with small flocculi—straps and roller re-applied. 28th. Two ounces of fetid fluid drawn off. From this time till the 8th Feb. small quantities of fluid were occasionally drawn off, fetid at first, thin and serous afterwards; the tumour decreased, its parietes thickened, and the child's health improved. On the 8th, the child was found to have pyrexia, with costive bowels. Aperients and antimonials were ordered, and in three days the child died in convulsions. The tumour was now only an inch above the level of the surrounding parts, and was nearly solid; the integuments were corrugated over it.

*Sectio Cadaveris.* " On making a division of the tumor, along the course of the spine, there was found to be a deficiency of part of the rings and spinous processes of the two last lumbar vertebræ, and the space had been filled up by newly-organized matter. The walls of the tumour, were from about half an inch to an inch and a half in thickness, in different parts of it. The cavity itself of the tumor was complete without any adhesions, and about two inches in diameter; its internal surface was very irregular, having a thickish coating of lymph upon it; besides which, two or three layers of organized matter could be peeled from the part beneath. Upon making a minute dissection of the neck of the tumour, two filaments of nerves could be traced through the newly-organized lymph from the medulla spinalis into the cavity of the tumor, as seen in the drawing, No. III.* The parietes of the tumor were condensed and thickened; and in the substance of the newly-organized matter were found little cavities, about the size of horse-beans or peas, containing a limpid fluid; and the skin covering the tumor was much corrugated and thickened. At the upper part of the tumor, immediately beneath the skin and cellular substance, were observed two or three strata of condensed membrane, about the thickness of a five-shilling piece; this is beautifully shewn in the drawing, No. IV.†; it had no doubt been the original boundary of the cavity of the tumor in which the fluid had been contained, and most probably was composed of arachnoid membrane and dura mater. From this original wall to the cavity of the tumor was the newly-organized matter, which, in different parts of it, was from half an inch to an inch and a quarter in thickness. On the right of this cavity there was another cyst, composed of a substance like thickened dura mater, about the size of a billiard-ball, quite impervious, and its internal surface smooth; the wall of this cyst could be traced into, and appeared to be a continuation of the external layer, or what would be the dura mater of the other tumor. This cavity was probably formed between the arachnoid membrane and dura mater, as on dividing the septum between the two, it was much thinner than the original parietes of the other cavity, and appeared to be bereft, as it were, of one layer of membrane.

Here we may observe that the process by which the soft parts become consolidated, is very gradual; before this tumor was punctured, its parietes were nearly as thin as a bladder, and were much distended, being fourteen inches in circumference. For the first three times after puncture the tumor became as much distended as before, and but little alteration of its walls was perceived; but upon the fourth, fifth, and sixth days, the parietes began to thicken, and at length to contract, and so on until the 11th of January, when the tumor was evidently much diminished in size, and the adhesive inflammation going on. From this time till the 1st of February, a gradual thickening of the parietes, and diminishing of bulk of the tumor, was taking place, when, in a few days, the child died of fever, and the appearance of the tumor was exactly the same as represented in the drawing No. II."‡ 41.

With respect to the treatment, and the mode in which that treatment operates, Mr. Stafford makes the following remarks.

"From the history and dissection of this case, as well as of those treated by Sir Astley Cooper and others, we may safely come to the conclusion that repeatedly puncturing the tumor, so as to let out the fluid as often as it

* Vide Drawing in the Library of the College of Surgeons.
† Ibid. ‡ Ibid.

collects, is the only mode at present known by which a permanent cure of spina bifida may be effected. Sir Astley Cooper used a needle for this purpose, though this method will not always succeed, as the fluid is so thick that it cannot pass through so small an aperture; under these circumstances it will be necessary to make a valvular opening in the parietes with a lancet, and when the fluid is evacuated, to close up the lips of the wound with adhesive plaster, so as to heal it up by the first intention. By this means also the air will be excluded, which is of the utmost consequence, for it has been observed that in almost all those cases in which the tumor has burst of its own accord, or the wound has not healed, the air being admitted, the patients have died very soon after;—a proof how necessary it is to use every precaution to prevent its entrance. After having evacuated the tumor, a soft linen compress should be laid upon it, and straps of adhesive plaster applied over this, so as to make gentle pressure, and a roller wound lightly round the body at first; and this pressure should be gradually increased till at length a truss may be applied, as in Sir Astley Cooper's cases. If, however, as frequently happens, convulsions are caused, or any other bad symptom produced, then pressure should be applied only to that degree the patient can bear. The tumor should be punctured every, every other, or every third, fourth, or fifth day, or indeed as often as the fluid collects; and it is advisable that this should be done before it arrives at its original size, for by the constant stretching of the skin it might lose its power of contractility, and the cure be retarded, or perhaps totally prevented." 42.

The general health of the patient should be attended to by general measures, dietetical and medical. The succeeding cases are intended to illustrate the palliative treatment and spontaneous cure of spina bifida. The palliative treatment, compression, produces general thickening of the parietes of the tumour, but exerts no further influence on its contents. The spontaneous cure is effected by the tumour becoming so large, that its distended parietes inflame, ulcerate in one or more parts, and so give issue to the contents. The walls then collapse—the aperture through which the fluid passes is closed by lymph and heals—the tumour again becomes distended and bursts—the parietes again collapse—and the wound heals again as before. This process is repeated as often as the tumour fills, and, during this time, coagulated lymph is thrown out from its internal surface, the walls become successively thickened, and at length they are consolidated. This process gave the hint on puncturing, which was adopted by Messrs. Abernethy and Cooper. But this remains to be observed:—when spina bifida is cured by art, hydrocephalus frequently ensues; when spontaneously cured in the manner described, Nature leaves an aperture covered by vesicles on the top of the tumour, and communicating with the canal. When fluid collects the vesicles burst, and it thus escapes, and obviates the danger of hydrocephalus. The rupture of the vesicles undergoes exactly the same process as the rupture of the tumour—they are distended, burst, and heal. It is a question whether, after spontaneous or artificial cure, the deficiency of bone is ever supplied by new osseous deposition. In general, it appears that it is not, and we possess no positive evidence to prove that it ever is so. We may now proceed to mention other cases.

*Case* 2. Alfred Church, aged two years and three months.

"There is situated on the lower part of the back, over the inferior lumbar vertebræ and superior portion of the sacrum, a tumor of firm consistence and of rather unequal surface, about the size of half an orange; it is moveable, and of a darker colour than the adjacent integuments; when pressed upon or moved, the fæces are instantly evacuated. The spine, above the tumor, is slightly curved outwards; the inferior extremities are singularly deformed, and can be twisted almost in any direction; the flesh is flabby. When sitting, the right leg rests on the outer or fibular side, and with the thigh forms nearly a semicircle; the left leg rests on the inner or tibial side, and the toes of both feet point to the right. The head is scarcely enlarged, and the skull is firmly ossified, except at the anterior fontanelle. His faculties are good, and he has only as yet cut six teeth. The testes still remain in the abdomen. The urine dribbles away as fast as secreted, and causes an excoriation of the prepuce and scrotum. He was born with an imperforate anus; an artificial opening was made into the rectum, through which the fæces are now voided. The nates are not, as usual, separated by a sulcus or division, but present a continuous surface. At the time of birth (which occurred between the seventh and eighth month) his mother states that there was a large soft tumor or bag occupying the site of the present tumor, and hanging considerably over the nates; his head was greatly enlarged (nearly as big as the rest of the body) and the integuments of the forehead hung over the face. The accoucheur made several punctures in the bag, and discharged much fluid; the contents were four times subsequently evacuated, at the interval of two or three days; the sac then sloughed off, and the fæces were evacuated through the ulcerated opening. The intestines could be seen through it. During the progress of the healing of this sore, much fluid constantly drained off for some months, and the size of the head gradually diminished, and now there are small vesicles on the top of the tumor, which occasionally break, and discharge a thin limpid fluid, and then heal. His mother attributes the malformation of his head and back to a fall she received when nearly three months gone with child, by which she struck the corresponding parts of her own body, and the deformity of the inferior extremities to her having been frightened, and thrown down, about a week afterwards, by a man who had a similar deformity of legs. After this she suffered greatly during the remainder of her pregnancy with severe pains in the head and back, and the weakness of her legs was so great as to prevent her standing.

This case is perhaps the most remarkable that ever occurred of spina bifida, as it combines in it almost all the facts of importance that have ever been recorded, both by the ancients as well as the moderns; first as to the immense size of the tumor;—secondly, as to its combinations; there was hydrocephalus of great magnitude—deformed limbs—deficiency of the nates, with imperforate anus, and diminished nervous power;—thirdly, as to the progress of ossification of the vertebræ; the rings were not only defective, but the bodies also, and the intestines could be seen through the tumor which communicated with them, as the fæces were evacuated through it;—fourthly, as to the subsidence of the hydrocephalus when the tumour sloughed off—the possibility of which fact has been doubted;—fifthly, as to the reason suggested by the mother for the origin of the disease;—sixthly, as to the spontaneous cure—another fact hardly credited;—and seventhly, as to its present state and the age of the patient." 51.

*Case* 3. May 4th, 1826. This child is four years and a half old, stout and healthy. Over the third lumbar vertebra is a tumour, equal in size to a pullet's egg, with rather a broad base, inflamed appearance, feeling solid, not diminishing on pressure, with a constant discharge of a watery fluid from an aperture situated on the top of it. The child suffers no inconvenience, and plays like other children. All that could be learned from the parents was, that the infant was born with a tumour like a bladder on the loins, that it grew, and in a few months burst. After this, the walls became thickened and hardened, and a watery fluid was constantly discharged from it. The child is now nine years old.

*Case* 4. ——— Potter, aged three weeks.

"There is a degree of disfiguration and swelling of the dorsal vertebræ and the integuments have ulcerated. The ulcer is as big as a shilling, but oval-shaped, and the surrounding integuments are purple. No spinous processes can be felt, and the transverse ones are widely separated, leaving a hollow cavity, over which the ulcerated integuments are stretched. During the first two weeks after the child's birth the inferior limbs were perfectly motionless, but since that period the toes have begun to move, and their motion has daily increased; so that if the sole of the foot be now tickled, it is very perceptibly felt. When the child was born, the mother describes the swelling to have had the appearance of a bladder filled with water. She did not perceive the precise time when the fluid was evacuated, but in a few days it appeared more flattened, and the ulceration then commenced. The child has all the appearances of health. The ossification of the occipital bone has been remarkably imperfect, not being larger than a shilling; so that the contents of the cranium, from their gravity and want of resistance, give the head a very oval form." 54.

The ulcer was dressed simply, and pasteboard applied to the head. The sore healed, and the spinal cavity became covered with a firm skin. Hydrocephalus now came on, and the child died with its usual symptoms. On dissection, the cranium was found very considerably increased in its dimensions, the ventricles so much distended with water, that in some places the medullary matter forming their walls was altogether wanting. The brain was very firm; on the choroid plexus was a fine hydatid. On examining the tumour of the back, the spinal canal was obliterated, excepting a small portion where the separation of the vertebræ commenced. The integuments were adherent.

We have dedicated more space to the consideration of this part of the volume, than practical men might, perhaps, deem necessary. But the nature and treatment of spina bifida are not so well understood as they should be; and possibly the information actually obtained is not universally diffused. Any little addition to our knowledge is, therefore, desirable on the one hand, and a free dissemination of it is useful on the other. The following short summary may be added.

"As the radical cure of spina bifida is always attended with some degree of danger, there are, perhaps, some cases where we should not be warranted in attempting it. If the tumor is small, and does not increase in size—if its parietes are not inflamed, and it is not painful—if the health of the patient be good, and he suffers no inconvenience from it—then, perhaps, the palliative treatment may be most judicious; but if, on the other hand, the tumor is increasing in size, or its walls are inflamed or painful, or the constitution is suffering from local irritation, the radical treatment for the cure of this disease is most advisable.

There are some cases where, from bad symptoms and a complication of diseases, there is scarcely any hope of cure; such as when they are accompanied with hydrocephalus, bursting of the tumor, and paralysis of the lower extremities. It is true that in two former instances a cure has been effected, but they may be almost considered as exceptions to a general rule. In these cases, where the patient cannot recover, the only course is to use palliatives, and to make the way as easy as possible to the grave." 56.

### ON THE INJURIES OF THE SPINE.

Much has been said on these injuries by experienced surgeons, and few in the ordinary pursuits of their profession fail to meet with cases of the kind occasionally. It is incumbent on practitioners to understand their nature and treatment. The chief injuries of the spine are concussion—fracture—dislocation.

*Concussion.* In complete concussion we have complete paralysis of the parts supplied by the nerves which arise below the affected portion of the medulla. In partial concussion the corresponding paralysis is partial, and in cases of this kind we find many singularities, if not anomalies. Of the possibility of meeting with such the surgeon should be aware. An explanation seems to offer itself in the supposition that the medulla may be more or less generally shaken, and in addition to this, that the origins of particular nerves may be more particularly injured. Thus occasionally there is paralysis of parts above the injury, as well as below it. This is explicable on the first supposition. Mr. Stafford mentions this case. A man received a violent kick from a horse on the most projecting point of the dorsal vertebræ. He was immediately paralysed in the lower extremities, and the arms lost all power of motion with partial loss of sensation. Under such circumstances, the upper extremities usually suffer less than the lower at the time, and recover with more rapidity. All the parts below the injury are not always paralysed; of this Mr. Stafford mentions an instance.

*Case.* A pack of goods struck a man between the scapulæ, at about the seventh cervical and first and second dorsal vertebræ; the arms were immediately and totally paralysed, and there was partial loss of power in the muscles of respiration. Neither the inferior extremities, the bladder, nor rectum were at all affected. Probably the origins only of the nerves supplying the arms were injured. We had under our charge at St. George's Hospital a case of partial paralysis from concussion of the spine.

*Case.* A young woman was flung out of window by her gallant. She was admitted into the hospital immediately afterwards, complaining of great pain in the dorsal and lumbar region, and loss of power over one lower extremity. Next morning there was loss of power of motion in the thigh, and numbness in the natis of that side. There was paralysis of the bladder, and great torpor of the rectum. Under cupping and antiphlogistic treatment these symptoms were in a few days nearly removed. The following is a curious case, though not fairly introduced under the head of concussion.

"Oct. 1831.—About eight years from the above date, a man, belonging to the town of Penkridge, in Staffordshire, fell from the top of a waggon-load of hay. He was taken up in a perfectly helpless state, and was immediately carried to bed: he had struck his back upon the second, third, and fourth lumbar vertebræ, which were considerably displaced laterally, the body leaning to the right side, leaving but little doubt that the spine at that part had suffered fracture. He was perfectly paralysed below the injury; the fæces escaped involuntarily, and the bladder could not expel its contents; the arms likewise were partially paralysed, in both the powers of feeling and motion. The treatment of the case I am unacquainted with, but he has kept his bed ever since, and his present state is as follows:—The muscles of the right arm are so contracted that it is closely fixed to the side; the forearm from the same cause, rests upon the humeral part; the wrist is bent on the forearm, and the fingers are firmly clenched in the palm of the hand; the sense of feeling also is partially lost; the left arm is affected in the same manner, but not in so great a degree; the right leg has both the power of motion and feeling; the left leg has the power of feeling, but not that of motion; the sphincter muscle of the rectum remains paralysed, the fæces still escaping involuntarily, and the bladder only expelling half its contents." 61.

We proceed to Mr. Stafford's description of the effects of concussion of the spine.

"The patient immediately after the injury, as in all cases where the nervous system has received a violent shock, becomes almost lifeless—a marble coldness pervades the whole frame, but more particularly the paralysed limbs. He cannot lie in any other position than on his back;—his pulse is weak and faltering; and he is frequently almost unconscious of what is passing around him. In fact the whole frame is in a state of collapse: in this situation he remains for a few hours, when reaction gradually commences. The warmth of the body returns until it amounts to a state of fever. The pulse rises, becoming quick, hard, and full; the tongue is furred, and great thirst is felt. The patient has more or less pain in the injured part, and sometimes delirium supervenes. If the injury be received high up, there is dyspnœa, in consequence of the muscles of respiration being paralysed, and, as I have before stated, the parts below suffer paralysis, according to where the injury was received.

Although the above symptoms commonly happen, yet they do not invariably occur; the pulse does not always become quickened, nor does it always become increased in fulness; it not unfrequently remains stationary without any perceptible change from the natural standard of health: it sometimes even becomes slower than natural, being reduced in beat as low as 60, or even as 40. The temperature of the body likewise does not always increase; the animal heat may be much the same as before the accident took place. In many instances also, if a thermometer be applied to the paralysed extremities, they will be found to be some degrees lower in temperature than those which do not suffer from the injury.

There are other symptoms besides the above consequent upon injuries of the spine. The bladder is not only paralysed, but the urine contained in it generally becomes decomposed, and fœtid and calcareous matter is frequently deposited. From this cause the mucous surface often inflames and ulcerates, by which the death of the patient is accelerated. This is a very curious fact; for it would appear from such a circumstance that the nerves of the organ had some influence over its contents. Thus, if from any cause the bladder loses its power, from a deficiency of nervous influence, the urine immediately upon being secreted from the kidney, undergoes, in some measure, that change which it would do when it has entirely passed from the body. It would seem therefore, that when the energy of this organ is diminished or entirely lost, that it resembles in some degree an inanimate vessel. It would not be possible to account for these symptoms unless the nerves had some power over the contents of this organ, which would prevent decomposition." 65.

This is not only an imperfect, but an erroneous description of the alterations which take place in the urine and urinary organs. From Mr. Stafford's observations the inexperienced reader would be led to imagine that all the changes in the urine occur in the bladder, from the loss of its regulating or antiseptic powers. Mr. Stafford does not mention the alkalescence of the urine, which does, however, in general, take place. Now the urine, in many cases at the very least, becomes alkaline or is secreted so in the kidney, and that this is the case in the present class of instances is proved by the fact that alkaline urine is obtained if a catheter be left continually in the bladder. When this is done no collection can take place in this receptacle, and the fluid is obtained as sent down from the kidney. The alkaline urine contains the triple phosphate, a salt which is well known to be formed in the kidney. The alkaline urine becomes, however, a source of irritation to the mucous membrane of the bladder, which falls into a state of chronic inflammation: adhesive mucus is then secreted, which, mixed with the triple phosphate, often constitutes a sort of alkaline glue at the bottom of the urinal. It is very probable that phosphate of lime may now be secreted by the bladder, increasing the alkalescence of the urine. These changes will readily be understood by those conversant with the morbid chemistry of this important excretion. We have not space to pursue the subject further, and we trust that at no distant period of time, the injuries of the spine will be treated of by one who is well able to do justice to any practical subject, we allude to Mr. Brodie. This distinguished surgeon has already favoured the profession with a paper on the primary effects of injuries of the head, but he yet owes us a debt which, importunate and clamorous though we may appear, we beg to remind him of—the sequence of that paper. In the mean time we correct Mr. Stafford on a point in which he has evinced some sins both of omission and commission.

Another symptom is torpor of the bowels, owing greatly, no doubt, to paralysis of their abdominal muscles, if not of their own: and flatulency, owing partly to the constipation, partly perhaps to diminution of the nervous influence by which the living gut controls the chemical decomposition of its contents. However this may be, the facts are as stated. Priapism is well known to be another occasional consequence of injuries of the spine. It has been attributed to concussion of the cerebellum, a gratuitous speculation.

The prognosis of concussion of the spine must depend on the seat of the injury, and its extent. The first is ascertained by inquiry and inspection, the second by the symptoms.

"The treatment of concussion of the spine must be adapted to the three different stages in which it presents itself to our notice. 1st. That immediately after the receipt of the injury; 2d. The inflammatory stage; 3d. That when the active symptoms have subsided. In the first stage, immediately after the receipt of the injury, the patient is usually so exhausted from the violence of the shock, that he is in a state of collapse. All that can be done at this time is, to place him on his back in a warm bed, and to administer stimulants, such as brandy, camphor, carbonate of ammonia, &c. until reaction commences. When reaction has taken place, antiphlogistic measures should be immediately resorted to. If the fever runs high, and the pulse be quick, full, and hard, the patient should lose blood from the arm. If, however, the symptoms are not sufficiently violent to demand very active treatment, he should be repeatedly cupped opposite the injury, purgatives also should be given, first to act briskly, then moderately upon the bowels. The feverish symptoms will be best allayed by the exhibition of antimonials combined with nitre, and small doses of saline purgatives. When the activity of the inflammation is diminished, blisters may be applied (and repeated as often as the preceding ones have healed) on each side of the spine, with the greatest advantage, until all inflammation has entirely subsided. During the treatment the patient should take nothing but farinaceous diet, and it is of the utmost consequence that the spine be kept as still as possible, for each movement, by increasing the inflammation, must necessarily diminish the progress towards cure. To effect this, the double-inclined plane-bed, invented by Mr. Earle, will be found of the greatest service, as when the patient is placed on it there will be no necessity for him to be moved, either to perform the natural evacuations, or to have those means employed which are necessary to the restoration of his health.

In concussion of the spine, the muscles of the rectum are usually either so paralysed that they have no power to act, or that the fæces escape without their being able to control them: in the former case injections must be used. The bladder likewise is unable to expel its contents. A catheter in most cases must be passed at least twice in the day, or as often as a sufficient quantity of urine is secreted.

It often happens that patients who have had bad concussions of the spine recover to a certain point, and yet do not get quite well; that is to say, they have either an imperfect power of motion, or an imperfect sensation. It is difficult to say exactly from what this arises, but it is most probable owing to chronic inflammation, or to some alteration of structure of the medulla, in consequence of the injury. It should be treated, however, according to the symptoms. If pain still exists in the injured part, the means employed should be the same as in diseases of the spine. The patient should constantly lie upon his back, counter-irritants should be used, by repeated blisters, the moxa or issues, and the general health should be attended to. If, however, there is no pain, and there is reason to believe that the defective power arises rather from weakness than from any other circumstance, then stimulating the part will be the most likely means of restoring the lost powers, and the best means for this purpose will be friction on the spine and limbs, stimulating liniments, shampooing, electricity, and galvanism, and if the power of movement alone be defective, then the limbs affected should be made to undergo certain exercises calculated to restore them." 72.

We would not recommend practitioners to be so much alarmed about the stage of collapse. It is with injuries of the spine, as with those of the brain, in this particular. If the injury is remediable the collapse is very seldom, very seldom indeed, so profound as to inspire serious and just alarm, or require stimulants which may afterwards be injurious. If the injury is so severe as to occasion a collapse that will prove fatal, that injury will kill in spite of all our cordials. We would not be mistaken. We do not affirm that patients *never* sink from collapse independent of lesions which must necessarily be mortal, but we affirm that they very rarely do so, and we confess that we never saw an instance of the kind. But we do say, that for collapse as it usually presents itself, no stimulating treatment is necessary. Warmth and the bed are all that are required, and if more active measures are unnecessary they are likely to be injurious. Mr. Stafford relates two cases; we shall take one. Previous to this we shall give the cadaveric appearances in those who die from concussion of the spine.

"The parts exterior to the osseous canal are much contused, and sometimes blood is found extravasated in the cellular membrane. The membranes of the medulla spinalis are in a high state of vascularity, opposite to the point where the blow took place, and occasionally one or more of them are ruptured. There is also more or less blood, either coagulated or not, between the dura mater and osseous canal, the dura mater and arachnoid membrane, the arachnoid membrane and pia mater, and sometimes between all at the same time. When the patient dies immediately after the accident, the medulla spinalis is generally of its natural colour and texture, but now and then it is rather redder, and if minutely examined through a magnifying glass, the capillaries ramifying upon it may be seen injected with blood. When the death of the patient has been delayed, it is not uncommon to find it of a yellowish red colour, and of a softer consistence, and in some cases where there has been much inflammation, it is rendered into a more fluid state at the part where the concussion took place. Occasionally it is observed that the inflammation of the injured portion has extended itself along its own substance to the brain, as well as along its membranes, to those of that organ, and in this case there is a quantity of fluid found in the vertebral canal." 79.

We cannot look upon the foregoing chapter on concussion, as either displaying all that is known on the subject of concussion of the spine, or even as evincing method and precision. After injuries of the spine we meet with two series or orders of symptoms; the one immediately consecutive to the injury, and expressive of its charater and extent, the other arising after a certain interval, and dependent on chronic inflammatory action set up in the medulla, its membranes, or even in the spinal column. These have not been well separated and discriminated by Mr. Stafford. But to pass to one of the cases.

*Case.* James Liddy, æt. 30, admitted into St. Bartholomew's Hospital Feb. 5th, 1830.

About the middle of January, after throwing himself from a wall 12 feet high, he immediately found a sensation of chilliness, pricking, and partial inability to move the right leg; he was likewise unable to make water or retain his fæces. Previously to his admission he had taken some medicines, which had been of no service. On his admission, he complained of considerable pain in the lumbar region, increased by tapping the spinous processes, and pain along the anterior and upper part of the thigh, in the inguinal and hypogastric regions, with some swelling in the groin. He was cupped and twice blistered, with such relief, that on the 13th he was able to leave the hospital. On the 20th he returned, with considerable uneasiness in the leg, thigh, and hip, with disposition to sickness and thirst. He was bled to 16 ozs. took laudanum in an effervescing draught, was blistered and cupped. After this treatment there remained but slight uneasiness in the right thigh and knee, with an inability to rest the heel on the ground. On the 9th March, a moxa was made in the lumbar region, and soon afterwards he voluntarily left the hospital. After this Mr. Stafford has made some mistake in the dates. It seems that he was again in the hospital, and left it in September, with the action of the bladder still imperfect, and a slight pain in the thighs. He now pursued his occupation—that of a corn-porter, when he soon became worse. In December, apparently, he returned, with complete incontinence of urine, some difficulty in retaining his motions, pain on pressure in the lumbar region, and also along the inner side of the thighs, with great tenderness on pressure. His general health was good, but he did not sleep well. He was put on milk diet, ordered to keep the horizontal posture, two moxas were applied to the lumbar region, and some physic given. The dribbling of urine, and feeling as if he wished to void it, continuing, a catheter was passed, and about a pint of healthy urine drawn off; there was a slight stricture about three inches from the orifice of the urethra. He was ordered to wear an elastic gum catheter plugged, and to have the water drawn off every four hours. On the 6th Dec. two moxas were again applied. On the 10th, he complained of great increase of pain in the thighs, extending also to the calves of the legs. Eighteen leeches were applied to the thighs with much relief. On the 18th, the pain in the legs was very severe, and the same application was made to them with some success. On the 1st of January, a blister was applied, with good effect, to the left calf for pain there. He could now bear pressure on the spine without pain, but complained of cold and shivering. On the 1st February he was in good health, but did not sleep well; the urine was passed three or four times daily, but only at the water-closet, and he always felt a desire to pass more; he introduced the elastic catheter every evening, and drew off half-a-pint of healthy urine; there was no difficulty in retaining the fæces. In this state he left the hospital, with the moxas not yet healed.

### FRACTURES OF THE SPINE.

Under this head we find nothing very new or instructive, but some cases are related which are worthy of extraction, for facts are the matériel of medical science. In order to shew that fracture of the spinous processes of the vertebræ is frequently recovered from, we have two cases related.

*Case* 1. "In the month of December, 1820, a soldier, belonging to the North Gloucester Militia, was at work in an excavated stone pit, when the roof gave way, and fell upon his back. He was immediately dragged by his fellow-workmen from the stones and mould which were upon him, and was carried home. He was examined, and it was found that the spinous point of the first lumbar vertebra was not in a regular line with the others; it was depressed, and fractured. The man had complete paraplegia below the injury, and the bladder and the rectum were also paralysed. When reaction was established he was bled largely, and powerful doses of purgative medicines were administered, in every form and dose, together with the employment of purgative clysters, but with no effect, until the eighth day, when the torpor of the bowels was overcome. He was now unable to retain the fæces, and they made their escape involuntarily. His bladder also still remained paralysed, and it was necessary to relieve it constantly by the catheter. He continued in this state for three months; at about which time sensation began to return, with pricking and shooting pains in the limbs, and shortly afterwards he perceived he could move his legs slightly backwards and forwards. In six months both these powers had so increased that he could walk with crutches, and in twelve he could go about without any support, and all that he suffered from was weakness in the limbs. The bladder and rectum gained their power also, in the same ratio as the other paralyzed limbs. When I last saw him, the parts over the injury were much thickened and consolidated. It is probable that there was more injury in this case than fracture of the spinous process; but this can only be proved by the examination of the parts after death.

*Case* 2. A man was admitted into St. Bartholomew's Hospital for an injury of the back, but he had no symptoms whatever of concussion of the spine. There was, however, an irregularity of the lower spinous processes of the dorsal vertebræ, and it was found that the tenth was flattened and depressed. The part was cupped and fomented, and aperients administered. In a month he went away quite well." 86.

Sir Astley Cooper has shewn that Nature is capable of repairing fractures of the vertebræ as of other bones, in fact, there can be little doubt that the fracture itself is of comparatively little moment; it is the implication of an organ so important as the medulla, which confers upon it the seriousness and danger which attend it. Mr. Stafford gives the brief particulars of two cases, calculated, in a high degree, to teach caution and gentleness in the handling of patients who have suffered from fracture of the spine.

*Case* 1. "July 25th.—A man having received a kick from a horse, upon the back of his neck, became instantly paralyzed in all parts of the body below the injury. He was immediately carried to St. Bartholomew's Hospital, and in about twelve hours after the accident delirium came on. The surgeon desired that the head might be shaved, in order that the proper remedies might be employed. In doing this, the barber turned the head a little on one side; the patient instantly fell from under his hand, and expired. Upon examination it was found he had fractured the third cervical vertebra, and that the sharp point of the fracture had pressed upon and wounded the medulla.

*Case* 2. A man fell from a great height, and broke his neck. An officious nurse, in moving him up, as she thought to relieve him, let his head fall backward. He instantly expired. He had fractured the second cervical vertebra.

Both of these cases occurred to my own knowledge, and from them it may be seen how necessary it is to keep the spine quite motionless, and more particularly when the accident happens in the neck. As soon as the patient receives the injury, he ought to be placed on one of the double-inclined plane beds, upon his back, and he should never, if possible, be moved, until there is sufficient reason to believe the fracture is united." 95.

We pass over some cases of fractured spine, and merely insert the morbid appearances observed after these injuries.

"When only simple fracture of the spine takes place, the morbid appearances are generally the same as in concussion; but if there is a complication of injuries, such as depression of the bone, &c. or there is dislocation of the vertebræ from one another, then it is found that compression, contusion, or either total or partial laceration of the medulla spinalis, may be produced. When the medulla is compressed, it does not always follow that there should be any difference from concussion in the morbid appearances, although most generally it is found that either some of the membranes are torn, or there is an extravasation of blood in the canal, and the medulla itself is much redder than usual. When the medulla spinalis is contused, it is usually found to be soft and disorganised; being occasionally streaked in its external substance with coagulated blood, and in one instance, mentioned by Sir E. Home, a clot of blood was found in its centre.

The membranes may be ecchymosed, ruptured, or have coagulated blood between them. If long after the injury, the medulla may be changed both in colour and structure, being in the one of a yellowish, red, or grey, and in the other much softened. Laceration of the medulla almost always occurs when there is fracture with dislocation, or when there is dislocation alone, and it may happen even under any circumstances, when there is displacement; the medulla, in these cases, may either be partially or wholly lacerated, and generally its membranes are torn, and blood is effused in the vertebral canal." 107.

The fourth chapter treats of dislocations of the spine. These are so rare, that Sir A. Cooper never saw an instance of the accident. When dislocation does occur, it is, from obvious reasons, more likely to take place in the neck than in any other part: elsewhere, indeed, it may be looked on as impossible. The most usual seat is between the atlas and dentata. J. L. Petit relates a case, which is also quoted by Boyer, where the only son of a tradesman went into his neighbour's shop, and playing with his infant son, lifted him from the ground, by putting one hand under his chin and the other on the back part of the head, by which he dislocated his neck, and the child instantly died. The father in his passion pursued the unfortunate homicide, and threw at him a saddler's hammer, the sharp point of which penetrated between the first and second vertebræ, and divided the medulla spinalis, and killed him upon the spot also; and thus, as he remarks, they both perished from nearly the same accident. Mr. Stafford relates a case which occurred at St. Bartholomew's Hospital.

*Case.* A bricklayer ascending a ladder, at a great height, lost his hold andfell upon his head. He was taken up dead, and conveyed to the hospital. On examining the spine, it was found that he had dislocated the first vertebra from the second, the ligaments of the processus dentatus being torn. Mr. Stafford relates three cases, to prove that dislocation of the cervical vertebræ, lower down, can and does take place; they are interesting, and deserve to be recorded. In all, the dislocation appeared to have been effected by the neck being bent forward to an extreme angle, so that the articulating surfaces were dislocated and the ligaments torn.

*Case* 1. "A man was working upon the roof of a house, and, having lost his balance, fell upon the pavement with his head foremost, and bent his neck forward to an extreme angle. He was taken to the hospital. Some displacement was observed between the fifth and sixth vertebræ; and the upper part of the column was protruded a little more forward than the lower. He had paralysis of all the parts below the injury. The usual remedies were employed, but he died in a fortnight. It was found that there was a proper dislocation of the fifth vertebra from the sixth; but that neither the articulating processes, nor the rim of the body of the vertebræ were fractured.

*Case* 2. A man also fell from a height, and bent his neck forward to an extreme angle. He died, and it was discovered that he had dislocated the fifth vertebra from the sixth, without fracture of any part of either of them." 113.

*Case* 3. Charles Butcher, æt. 22, Jan. 8, 1827. Is a stout muscular man, and, while descending a step about two feet in height, with a barrel (1 cwt.) upon his shoulder, he fell upon his buttocks, the barrel resting on the back of the head and upper part of the neck. He was brought to the hospital forthwith. He was perfectly sensible—there was total loss of sensation and voluntary motion below the neck—respiration performed solely by the diaphragm—pulse weak and slow—body cold—priapism. He was placed on his back in bed, and, there being reaction in the evening, he was bled to 16 ounces, and a dose of calomel and jalap was prescribed; four ounces of urine were drawn off by the catheter. On the 9th, he complained of pain in the lower part of the neck—respiration slower—slight power of motion in the arms—a little feeling in front of the chest—pulse full—heat diminished—fæces dark and offensive and passed involuntarily—a catheter introduced, but only a table-spoonful of urine drawn off; in the evening six ounces of high-coloured urine were abstracted. He slept during the night and felt better next day. The pulse and temperature of the skin remained the same—there was a tingling sensation in the hands, and sensation in the upper parts of the arms and thighs—priapism continued more or less—the fæces had been discharged involuntarily—and he complained of distention of the bladder. Eighteen ounces of high-coloured urine were drawn off; it deposited on cooling a small quantity of dark brown sediment. On the

11th he was worse—the countenance unfavourable—tongue brownish, and dry in the middle—fæces passed involuntarily—slight sensation still in arms and legs—thought he could walk home and asked for his clothes to dress himself. Six ounces of lighter coloured urine were drawn off. After this he sank rapidly, the priapism continuing, and died on the morning of the 12th. On examination there was found complete dislocation of the fourth cervical vertebra, its inferior oblique processes having passed in front of the superior oblique processes of the fifth; its body, separated at the fibro-cartilage, stood over that of the fifth by its whole depth. No mention is made of the state of the medulla or of other parts.

The succeeding chapters from the fifth to the tenth, including both, are occupied with the consideration of the diseases of the vertebral bones and intervertebral cartilages. After the many excellent monographs which have been published, it cannot be supposed that Mr. Stafford could offer much novelty on such a subject. He has not, and all we can do is to select here and there a passage conveying some new view, or interesting fact. We will take the following remarks on the cancellous structure of the spinal bones.

"It may be observed, that upon breaking the substance of the body of a vertebra in a fresh subject, the cancelli are found to be composed of thin transparent plates, and of round pillars of bone, both of which arise from the internal surface of the external plate surrounding the body, and are irregularly disposed and connected one with the other, throughout its whole substance. These plates are slightly transparent, and between their interstices is deposited an oily unorganized matter, which answers to the marrow in the long bones. When the phosphate of lime is dissolved by diluted nitric or muriatic acid, the bone is rendered soft, and the cancelli become quite transparent: they still retain their reticulated appearance, but their substance is so pliable that it resembles sponge, and can be compressed into a very small compass. Upon a closer examination of the cancelli in this state, they are found to be of a membranous structure, having a smooth, shining surface, on each side of them, with rounded edges, and being of a dense, tough consistence. Hence it would appear, from the common character of membranes being smooth on one side and rough on the other, that they are composed in their natural state of a duplicature of membrane, with phosphate of lime either deposited in their texture or on their surfaces.

Having so far shown by this description that the cancelli are of a membranous structure, I shall now endeavour to prove that they resemble other membranes in their functions as well as in their diseases. They possess the power of secretion as other membranes; for instance, they secrete phosphate of lime, and the oily matter deposited between their interstices. That the phosphate of lime is a mere secretion, is proved from the fact, that its quantity is increased or diminished according to the healthy or unhealthy state of the membrane, as may be seen from its deficiency in rickets and scrofula. In rickets the earthy matter is almost entirely absorbed, and the cancellous membranes are left uncovered by it. The same takes place in scrofula; the bone becomes softened, that is to say, the action of these membranes is changed; they do not secrete phosphate of lime, but an albuminous cheesy matter. In rheumatism the contrary takes place: there is more earthy matter secreted than ought to be, and the bone is rendered hard. These facts are illustrated by many preparations preserved in museums. The membranes of the cancelli also secrete an oily matter, which is lodged between the interstices. This oily matter is frequently entirely wanting in affections of the spongy texture of bones, and in scrofula its place is supplied by an albuminous or caseous matter, (a secretion peculiar to this disease,)

which proves that the action of this membrane is altered, and that the natural secretion must have been previously absorbed.

Although the membranes of the cancelli no doubt are of a character *sui generis*, yet from the diseases which affect them they appear more to resemble the serous and synovial membranes than any. Both of these are liable to scrofula; so is the cancellous structure, and the matter secreted is the same in all. They are also affected by rheumatism, by the action of mercury, and by the venereal disease: so are the membranes of bone. A greater proof, however, of their resemblance is, that serous membranes sometimes secrete phosphate of lime. As a proof of this, I have frequently inspected bodies where I have seen bony matter deposited on the dura mater of the brain, the arachnoid membrane of the spinal marrow, on the pleura, pericardium, peritoneum, &c." 120.

We think it very probable that a membrane does exist in the cancellous tissue of short and flat, and the cancellous extremities of long bones, although its existence has been denied, if we remember right, by Bichat. But we think the analogy between this membrane and the synovial is too loose; it is more allied to the medullary system of the long bones, a system sui generis. That bone is secreted by it, or rather by the vessels which it supports, is very likely, and as rickets consists of a diminution of the secretion of the phosphate of lime, that disease may be deemed in some sort an affection of this membrane. But rickets is undoubtedly a constitutional disease, and that it is not essentially a spinal complaint, as Mr. Stafford imagines, is proved by the rickety alterations of other, and especially of the long bones.

Our readers are, perhaps, aware, that abscesses are occasionally found in the ends of long bones, as the tibia, and very serious complaints they are. Mr. Brodie has once or twice trephined the bone for symptoms of this complaint, and evacuated the abscess with success. But this by the way. There is a preparation of abscess in the substance of the bone of the sacrum, in the museum of St. Bartholomew's Hospital. The history of the case is unknown.

Ulceration occasionally takes place in the substance of the bony arches of the vertebræ. Practitioners should be aware of this fact.

"Although it does not commonly happen, yet in some cases ulceration of the rings of the vertebræ takes place. There is a preparation of this species of the disease in the spine in the museum of St. Bartholomew's Hospital. Here the ulceration began in the back part of the rings of the dorsal vertebræ, and a portion of the bone is separated, and pressing upon the medulla, while the bodies are perfectly sound.

In the museum of Guy's Hospital also, there is a preparation where an abscess is connected with the vertebral canal. It appears in this case that the rings of one of the dorsal vertebræ is ulcerated, from which there is a fistulous passage leading from the vertebral canal to a large abscess in the back. This narrow passage is partly filled up by caseous matter, which proves it to have been a scrofulous abscess, arising in consequence of diseased bone, and connected, owing to the ulceration of the ring, with the vertebral canal." 149.

An interesting case of complicated luxation, from disease of the atlas and dentata, has been given to our author by Mr. Lawrence, and is related under the head of disease of the articular joints. If we are not much deceived, this case was published by Mr. Lawrence in the Medico-Chirurgical Transactions, and noticed at the time in this Journal. We will not, therefore, again refer to it. From the chapter on angular curvature of the spine, we only think it necessary to make the following extract, illustrative, we imagine, of the providential solicitude of Nature to guard her children, even in the midst of distempers and diseases. The good effects resulting from the following constitution of parts, during and after a morbid process, are sufficiently apparent.

"With regard to the curvature of the spine, it may be considered as a general law, that the medulla takes its course along the greater angle; that is to say, it follows the curve of the rings of the vertebræ rather than that of the bodies. It may be observed, also, in most of the morbid specimens of this disease, that the vertebral canal, where the curvature takes place, is even larger than natural, and that the projecting points of the broken-down vertebræ are absorbed, and rounded off. In some cases, no doubt, where only one or two bodies are rapidly destroyed, and the angle is very acute, the bones may press upon the spinal cord, but in the majority they do not, and even in those where they do, if the patient lives long enough, there is but little doubt they are in general gradually smoothed down by absorption.

The state of the medulla itself, if examined after death at the part where the curve takes place, varies; sometimes it does not in any way deviate from health, the structure, both of the medulla and its membranes, is natural, while at other times a considerable degree of disease may have gone on. The membranes may be thickened, matter may be formed pressing upon them, or between them, and the medulla itself may be reddened, or partially softened, or softened in such a manner as to be almost in a fluid state. In these cases the paralysis below is usually complete." 167.

After enumerating the symptoms usually observed in ulceration of the vertebræ and intervertebral cartilages, Mr. Stafford properly observes that we must not be surprised at their absence in cases considerably advanced. There is no novelty in this remark, but there is truth, and truth so serious as to merit great attention on the part of practitioners. We recollect a striking instance of the fact, of which, indeed, we have seen several. A young man complained of some obscure pains about the hip-joint, without other evidence of organic disease in that articulation. Struck with this circumstance, we examined the spine very carefully, but detected no irregularity, no decisive tenderness on pressure, no appreciable functional disturbance. The patient had a fistulous opening in the integuments, under the right pectoral muscle, which was concluded, from its aspect, to lead to carious or dead rib, though no exposed bone could be felt with the probe. He was attacked with erysipelas around this ulcerated opening, and symptoms of latent pneumonia supervened about the fourth or fifth day. In thirty-six hours he died. On dissection, recent pleuro-pneumonia of the right side of the chest was found to have been the immediate cause of death. As had been suspected, two or three ribs were partly carious, partly dead. But this was not all. Between these carious ribs and the pleura costalis, which was thickened, chronically inflamed, and separated from the side, was a widely-diffused abscess. This passed backwards to the dorsal vertebræ, a great many of which (we now forget the precise number) were more or less ulcerated, with correspondent affection of the intervertebral cartilages. The hip-joint, to which the sympathetic pain had been referred, was healthy. We may insert some other instances of a similar description related by Mr. Stafford.

"Notwithstanding, however, all the symptoms here enumerated, so insidious is this disease that I have sometimes known angular curvature to occur without a single indication of it, as far as the health was concerned. A child, for instance, shall play about as usual, without its parents remarking any change in it; it shall have a good appetite, sleep well, &c. and yet ulceration of the bones shall be going on, which shall not be discovered until anchylosis of the part has taken place. I recollect a case of this description happening in a little boy of my own acquaintance. He had always been from his birth more lively and active than usual, and never had any illness to speak of. By mere accident his father discovered that he had an angular curvature of about two or three of the vertebræ, which had firmly anchylosed.

The completeness and incompleteness, also, of the symptoms, very much depends upon the rapidity with which the curvature takes place. If the destruction of the bodies of the vertebræ has been very quickly effected, the paraplegia is usually more complete; but if it has been slow in its progress, the paralysis below is frequently very imperfect. It is only astonishing in some cases how much mischief may occur to the vertebræ, both as to the extent of the curve and the acuteness of it, without the symptoms of pressure upon the medulla being produced. I have frequently seen six or eight of the bodies of the vertebræ destroyed without a single symptom as to the loss of the power of motion or feeling to denote it. I have at this present time a case of a child under my care, in St. Mary-le-Bone Infirmary, where at least six of the bodies of the dorsal vertebræ have been destroyed by caries, and the angle of the curve is very acute, and yet no paralysis has ever occurred. Such a phenomenon can only be explained by the very progressive and slow manner in which the alteration of form takes place." 185.

The directions for the treatment of the disease are judicious; but as most of the items are familiar to well-informed practical surgeons, we think it necessary to make but one extract. It shews that, possibly, the horizontal posture may be too assiduously maintained, or, at all events, that the injudicious extension sometimes resorted to by medicasters and quacks is not altogether devoid of danger.

"As the curative process by which the destruction of the bodies of the vertebræ, and that of the intervertebral substances, can only be effected by the anchylosis of the superior with the inferior portion of the spinal column, it may be advisable, perhaps, to assist the diseased parts in anchylosing. Instances have been known, one of which is shewn in a preparation in the Museum of St. Bartholomew's, where several bodies of the vertebræ have been destroyed, and where the two diseased extremities have not come in contact with one another, so that a large gap has been left between them unoccupied. This has been attributed by some (and it certainly is a very rational conjecture), to the patient having lain too long in the horizontal position; thus it has been impossible to approximate the two extremities. Whether this really is the cause, or whether it arises from the rings of the vertebræ and their articulations having sufficient strength to bear the weight they have to sustain, is uncertain. From whatever cause, however, it may arise, it appears to point out the necessity of our assisting the anchylosis of the two separate parts, which can only be effected by gradually allowing the upper part by its own weight to fall upon the lower part of the vertebral column. The best method to accomplish this is, to raise the superior part upon which the body or trunk lies, of the double-inclined plane bed, to rather beyond that angle which would be equi-distant between the perpendicular and horizontal line. By this plan the diseased surfaces will be approximated, and at the same time will be kept in a perfect state of quietude; thus the process will be going on without disturbance; by which the disease is cured." 202.

Nearly fifty pages are devoted to the consideration of distortions of the spine, lateral and otherwise. The reader would almost be tempted to suppose that such a subject was exhausted. It is true Mr. Stafford does not deal in novelties, nor could he; but what he advances is judicious. The last portion of the volume is occupied with the Diseases of the Medulla and its Membranes. It is generally lamented, and with too good reason, that few are sufficiently conversant with either the natural or unnatural appearances of these parts. We trust that, in spite of the obstacles which interfere with their inspection, the reproach will not be a perpetual one.

Mr. Stafford draws a parallel between arachnitis and tetanus, at least idiopathic tetanus; he seems almost inclined to consider them identical. With this sentiment we cannot agree, for two reasons;—first, we know too little of the symptoms of one disease, and morbid characters of the other, to be justified in pronouncing decidedly on their affinity—secondly, arachnitis does certainly occur, unattended by any tetanic symptoms whatever—and, thirdly, tetanus proves fatal, and yet leaves no traces of arachnitis after death. Of the latter assertion we need bring no proof. In support of the former, we need only mention one case which we recently witnessed at St. George's Hospital. A man had compound fracture of the skull, for which he was trephined. Hernia cerebri followed—it increased—a state of partial stupor, with some increase of pulse and heat of skin ensued, and he died. On dissection, there was found, with other things, inflammation of the arachnoid and pia mater of the brain, and continuous inflammation of those membranes investing the medulla. Over the latter there was decided effusion of coagulated lymph, &c. This patient had evinced no tetanic symptom whatever. It is right to mention that four cases are related by Mr. S. in three of which the spinal arachnitis was unequivocal, and in the fourth doubtful. In the first of the three, there were distinct symptoms of traumatic tetanus; in the other two, there were also tetanic symptoms occurring idiopathically. In the fourth, there was tetanus after burn. We give Mr. Stafford's hypothesis all the benefit it can obtain from the foregoing cases; but we venture to observe that they are too few and inconclusive to confirm his opinions.

### Hydro-Rachitis, or Dropsy of the Medulla Spinalis.

This arises from secretion of fluid from the spinal arachnoid, which gradually collects till it fills the whole canal. Its progress is usually very slow —its causes obscure—its symptom, paralysis, gradually developed—its treatment rather regulated by general principles than by experience. Mr. Stafford witnessed the following case in St. Bartholomew's Hospital.

*Case.* Thomas Cox, light porter, æt. 23, admitted Sept. 30, 1830.

"This patient, who is of moderate strength and stature, has usually enjoyed good health, with the exception of occasional headaches, which have troubled him from childhood. He has been accustomed to indulge pretty freely in liquors; but since his marriage, which took place a twelvemonth back, he has not, on an average, got drunk more than once a week. For three or four weeks previous to admission, he experienced uneasy sensations in the lower part of the belly, and a numbness of the thighs, accompanied with swelling of the right testicle. On Saturday evening, Sept. 25, he walked to the hospital to have his water drawn off, having never found any difficulty in passing it before. He afterwards walked home, a distance of half a mile, without difficulty, though he did not feel altogether firm upon his legs. He slept that night as well as usual, and found to his surprise, on Sunday morning, that there was complete loss of motion, and partial loss of sensibility of the lower limbs, with inability to make water. The fæces came away involuntarily in the course of the day; and the paralyzed limbs were occasionally affected with spasms, of which he was conscious. He was not feverish, and had neither head-ache nor other pains. On Monday he was cupped on the back by order of a Dispensary-Surgeon, who supplied him with medicines, and drew off his water till Thursday, when he was received into the Hospital, having derived no relief from the treatment." 274.

Cupping, leeching, blistering, warm-bath, the catheter, &c. were the means employed, but on the 5th the patient was worse; there was great weakness, furred tongue, dry skin, and feeble pulse. An alkaline calumba draught was prescribed, and a moxa applied on each side of the spine. The integuments covering the sacrum sloughed from pressure, the urine became loaded with ropy mucus, and offensive, and in spite of wine and broth the debility increased. A communication was now established between the bladder and rectum, its formation being preceded by the discharge of a small portion of sloughing membrane from the anus. Great emaciation ensued, with hectic cough, and on the 20th of January the patient died.

On examination, the arachnoid membrane, covering the upper surface of the brain, was raised by thin serous effusion, and the same kind of fluid was discovered under this membrane in the vertebral canal. The kidneys and ureter were healthy, the mucous membrane of the bladder dark-coloured, with the perforation of the viscus already alluded to. The legs were much swollen from anasarca.

Our author relates a case of effusion of blood into the spinal canal, independent of injury inflicted. He also cites a case which has been previously published by Sir Astley Cooper, and another by Mr. Chevalier. We shall take only the first, because it is original.

*Case.* J. Connell, æt. 31, was admitted into the St. Mary-le-bone Infirmary, May 23, 1828, with the secondary symptoms of syphilis. Mercurial ointment was prescribed, but, on the third night, having great dyspnœa, the ointment was discontinued, a blister applied on the sternum, and an aperient given. By these measures he was relieved, but he was seized in the night of the 26th with an attack of complete paraplegia; with severe pain in the dorsal region: pulse 80, sharp and full; bowels costive; retention of urine. He was cupped to 16 ounces, placed in the warm-bath, and the catheter directed to be used night and morning.

June 1st. Since last report has usually had hot skin, pulse above 100, sleeplessness, obstinately costive bowels; paraplegia still complete. He gradually grew weaker, a slough formed on the nates, the fæces were discharged involuntarily, and the catheter required to be passed twice daily, and on the 11th he died.

On examination, all the viscera were healthy, excepting the mucous membrane of the bladder, which was inflamed. A large quantity of blood was extravasated between the arachnoid and pia mater of the cord; it was semi-fluid, and filled nearly the lower part of the canal. The medulla was rather softer than natural in the dorsal region.

In Sir Astley Cooper's case the effusion was between the theca and bony canal; this case appeared to date its commencement to injury. The other case was that of a young lady, who had suffered no injury. She first complained of pain in the head and back upon the 26th of February, and died on the 4th of March. The extravasation was in the lumbar region.

"A child of 12 months old, who had just recovered from the operation for hare-lip, was carried out by the nurse. On its return home, it seemed in much pain, and appeared to have lost the use of its lower extremities: it died in three days. On opening the body I found the spinal canal full of a bloody serum, which, I have no doubt, was occasioned by slight extravasation from a strain, and subsequent inflammatory effusion. And how destructive such extravasation and inflammation combined, may be to the structure and office of the nerves, was illustrated by the case of a miller, who suddenly lost the use of his lower extremities, by lifting a heavy sack of flour. He died on the 15th day after the accident. On examining the vertebral canal, some extravasated blood was found mixed with a sanious matter, the theca vertebralis was evidently inflamed, and the nerves of the cauda equina were more completely rotten than I have found them after many weeks' maceration in putrid water, when removed from the dead body." 288.

We shall conclude this article by an instance of ramollissement of the medulla, which was witnessed by Mr. Stafford at St. Bartholomew's Hospital. It illustrates the general character of the symptoms.

*Case.* "Rebecca Dixon, æt. 16, admitted into St. Bartholomew's Hospital, November 1st, 1827: said that she had been struck with the handle of a mangle on the back part of the neck, in February last; but the injury was so slight that she took no notice of it until the beginning of September, a space of six months, when she applied to some public institution, on account of a severe pain in the upper and back part of her neck, and her face being drawn towards the right side: she took a number of remedies without benefit.

When she was admitted, she complained of severe and continued pain in the upper and back part of her neck; her face was directed over the right shoulder; she had not the least power of moving her head from side to side, and the least attempt made by another person to move it in that direction, produced excruciating pain. There was no defect either in motion or sensation of the extremities at this time. No disease of the vertebral column could be detected by an attentive examination. Her pulse was quick and feeble; her bowels were torpid, and required large and frequently repeated doses of aperient medicines to keep them free. Neither her respiration, nor her voice, were much affected at this time. She had never menstruated; her countenance was bloated and pale, without being particularly anxious. Her bowels were cleansed with calomel and jalap; and under the supposition that these symptoms arose from uterine irritation, the preparations of iron were given, with the occasional abstraction of blood from the back of the neck by cupping. This plan was persevered in during three weeks without benefit. About this time she had an attack of pain and tenderness in the abdomen, more particularly towards the right side, attended with vomiting, which continued till her death. The pain and tenderness were relieved in three or four days, by the use of leeches, and the exhibition of calomel and opium, the state of the pulse not justifying the use of the lancet.

Nov. 29th.—About this time she first perceived an inability to hold any thing small in her hands, and a sensation of numbness over the whole body, and an inability to stand or walk. These symptoms gradually increased, till they amounted to a total loss of sensation and voluntary motion: during the last two days of her life she passed her urine and fæces involuntarily: her muscles were rigid, and her limbs could be put in any position without pain. She died on the 11th of December." 296.

On examination, the spinal cord was found in a state of ramollissement about an inch below its commencement; it was pulpy, and stuck to the finger when touched, was of reddish-brown colour, and exhibited no traces of its original structure; this change occupied about an inch of the cord, the remainder being healthy. There were tubercles at the apex of each lung, adhesions on the convex surface of the liver, ulceration of the mucous membrane of the lesser curve of the stomach and of the intestines.

We have selected from this work such portions as appeared to offer any novelty, instruction, confirmation or otherwise, of unsettled points. It might be much condensed, and more attention might be paid to its literary composition. We meet with instances of defective grammatical construction, and such vulgarisms as, "it stands to reason." Faults of this kind are serious only because remediable with care and circumspection on the part of the author. Mr. Stafford is active, industrious, intelligent, and we wish him all success.

